# Multiple Sex Chromosomes and Evolutionary Relationships in Amazonian Catfishes: The Outstanding Model of the Genus *Harttia* (Siluriformes: Loricariidae)

**DOI:** 10.3390/genes11101179

**Published:** 2020-10-10

**Authors:** Francisco de M. C. Sassi, Geize A. Deon, Orlando Moreira-Filho, Marcelo R. Vicari, Luiz A. C. Bertollo, Thomas Liehr, Ezequiel Aguiar de Oliveira, Marcelo B. Cioffi

**Affiliations:** 1 Laboratório de Citogenética de Peixes, Departamento de Genética e Evolução, Universidade Federal de São, Carlos, São Carlos, SP 13565-905, Brazil; francisco.sassi@hotmail.com (F.d.M.C.S.); geizedeon@hotmail.com (G.A.D.); omfilho@ufscar.br (O.M.-F.); bertollo@ufscar.br (L.A.C.B.); mbcioffi@ufscar.br (M.B.C.); 2 Departamento de Biologia Estrutural, Molecular e Genética, Universidade Estadual de Ponta Grossa, Ponta Grossa, PR 84010-330, Brazil; vicarimr@yahoo.com.br; 3 Institute of Human Genetics, University Hospital Jena, Jena 07747, Germany; 4 Secretaria de Estado de Educação do Mato Grosso—SEDUC-MT, Cuiabá, MT 78049-909, Brazil; ezekbio@gmail.com

**Keywords:** cytogenetics, ribosomal DNA, comparative genomic hybridization, neotropical fishes

## Abstract

The armored *Harttia* catfishes present great species diversity and remarkable cytogenetic variation, including different sex chromosome systems. Here we analyzed three new species, *H. duriventris*, *H. villasboas* and *H. rondoni*, using both conventional and molecular cytogenetic techniques (Giemsa-staining and C-banding), including the mapping of repetitive DNAs using fluorescence in situ hybridization (FISH) and comparative genomic hybridization (CGH) experiments. Both *H. duriventris* and *H. villasboas* have 2n = ♀56/♂55 chromosomes, and an X_1_X_1_X_2_X_2_ /X_1_X_2_Y sex chromosome system, while a proto or neo-XY system is proposed for *H. rondoni* (2n = 54♀♂). Single motifs of 5S and 18S rDNA occur in all three species, with the latter being also mapped in the sex chromosomes. The results confirm the general evolutionary trend that has been noticed for the genus: an extensive variation on their chromosome number, single sites of rDNA sequences and the occurrence of multiple sex chromosomes. Comparative genomic analyses with another congeneric species, *H. punctata*, reveal that the X_1_X_2_Y sex chromosomes of these species share the genomic contents, indicating a probable common origin. The remarkable karyotypic variation, including sex chromosomes systems, makes *Harttia* a suitable model for evolutionary studies focusing on karyotype differentiation and sex chromosome evolution among lower vertebrates.

## 1. Introduction

Siluriformes (Actinopterygii; Teleostei) is a monophyletic order that covers a large deal of the freshwater fish diversity, with more than 3000 species and 36 families [1,2]. Of the six Loricariidae subfamilies currently recognized, Hypostominae and Loricariinae are the most representative ones, with the highest number of species (579 and 302, respectively) and a remarkable karyotypic diversity [2,3]. Loricariinae fishes are distributed throughout South and Central American rivers, including two tribes: Harttiini and Loricariini. The genus *Harttia*, popularly known as armored catfishes, is represented by 27 valid species [2,4].

Despite such great species diversity, cytogenetic studies are available for only eight of them, all from Brazilian Southern regions, with exception for *Harttia punctata* [3,5,6,7,8,9,10,11,12,13]. However, although still limited, the chromosome data are already enough to notice a conspicuous variation of the diploid number (2n) in the genus, ranging from 2n = 52♀/53♂ in *H. carvalhoi* [8] to 2n = 62♀♂ in *H. absaberi* [9]. Chromosomal breaks and rearrangements as Robertsonian fusions, fissions, as well inversions were proposed to occur in the karyotype diversification of *Harttia* species [13]. In addition, two multiple XY-derived sex chromosome systems were also identified: ♀XX/♂XY_1_Y_2_ in *H. carvalhoi* [8] and ♀X_1_X_1_X_2_X_2_/♂X_1_X_2_Y in *H. punctata* [12].

In recent years, a range of molecular cytogenetic investigations (e.g., repetitive DNA mapping based on fluorescence in situ hybridization (FISH), comparative genomic hybridization (CGH) and whole chromosome painting (WCP) has been applied in different fish groups, providing new insights into the evolutionary relationships among them [14,15,16,17,18]. However, the ribosomal genes are the only repetitive DNA class that has been analyzed in *Harttia* till now [3,8,12,13]. The rDNA mapping shows that the 18S sites presents a conserved pattern in number, with a single chromosome pair carrying these sequences in all analyzed species, but with variation in location and chromosome carrying them [13]. Similarly, the 5S rDNA is also found in a single chromosome pair in most species, with exception for *H. carvalhoi*, in which signals occur in both chromosome pairs 03 and 23 [8,13]. Besides, WCP analyzes are confined to only one species, *H. punctata*, highlighting the main chromosomal rearrangements involved in the origin of the X_1_X_2_Y sex system in this species [12].

In the most updated phylogenetical reconstruction based on mtDNA, three distinct clades can be recognized for the genus *Harttia* involving: (i) species that inhabit rivers of Guyana shield; (ii) species occurring in north Brazil, especially in Amazonas river basin and its tributaries and (iii) species distributed throughout southeast/south Brazilian rivers, such as Grande, São Francisco and Paraná river basins [19]. However, despite largely widespread in South America, only *Harttia* species from southern rivers have been cytogenetically studied to date. Here we analyzed three new species sampled from Northern Brazilian river basins by performing an extensive cytogenetic investigation using conventional cytogenetic protocols (Giemsa-staining and C-banding) combined with molecular cytogenetic ones, including CGH experiments and mapping of several repetitive DNA classes using FISH. The results allowed us to open a new chapter on the evolutionary history of *Harttia* fishes, besides describing new sex chromosome systems.

## 2. Materials and Methods

### 2.1. Sampling

**Figure 1:**
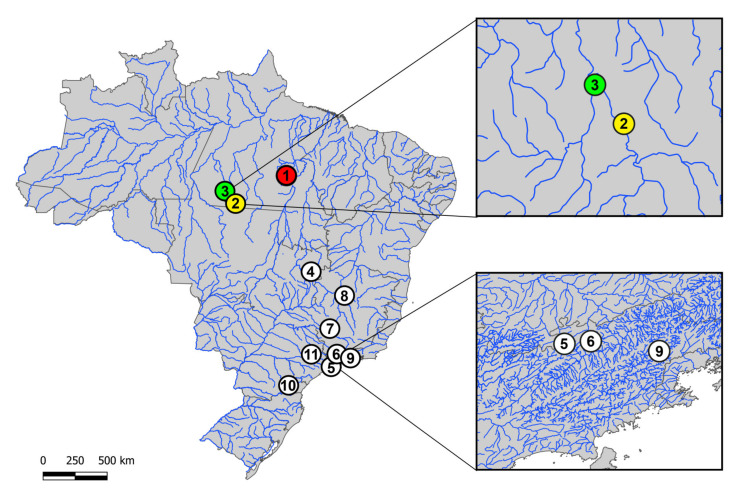
Brazilian collection sites of the three *Harttia* species cytogenetically investigated in the present study (colored circles) and the ones previously cytogenetically analyzed (white circles: data from [9,13]). **1**. *H. duriventris* (red circle); **2**. *H. villasboas* (yellow circle); **3**. *H. rondoni* (3-green circle); **4**. *H. punctata*; **5**. *H. gracilis*; **6**. *H. carvalhoi*; **7**. *H. torrenticola*; **8**. *H. longipinna*; **9**. *H. loricariformis*; **10**. *H. kronei*; **11**. *H. absaberi*. The boxes highlight the non-sympatric distribution of some species.

The collection sites, number and gender of the specimens investigated are presented in Figure 1 and Table 1. Samples were collected with the authorization of the environmental agency ICMBIO/SISBIO (License nº 48628-2) and SISGEN (A96FF09). The specimens were proper identified by evaluation of their meristic characters by Dr. Osvaldo Takeshi Oyakawa, curator of the Museu de Zoologia da Universidade de São Paulo (MZUSP) and specialist in this fish group. Samples from *H. punctata* were used for gDNA extraction and CGH experiments.

### 2.2. Chromosome Preparation and C-Banding

Mitotic chromosomes were obtained by the protocol described in [20]. Briefly, the animals were first injected in the abdominal region with a 0.025% aqueous solution of colchicine at a dose of 1 ml/100 g of weight. After 50–60 min, the specimens were euthanized, and the chromosomal preparations were obtained from cells of the anterior kidney. The experiments followed ethical and anesthesia conducts and were approved by the Ethics Committee on Animal Experimentation of the Universidade Federal de São Carlos (Process number CEUA 1853260315). C-positive heterochromatin (C-banding) was identified according to [21]. Briefly, slides were treated with 0,2 N HCl for 10 min, followed by a short wash in water; incubated in 5% Ba(OH)_2_ for 3 min at 42 °C ; rinsed in water for 1 min and then incubated in 2× SSC (pH 7.0) for 1h at 60 °C. The slides were then stained in 5% Giemsa solution (phosphate buffer, pH 6.8). 

### 2.3. Fluorescence In Situ Hybridization (FISH) for Repetitive DNA Mapping

The 5S rDNA probe included 120 base pairs (bp) of the 5S rRNA codificant gene and 200bp of a non-transcribed spacer (NTS) isolated according to [22]. In its turn, the 18S rDNA probe contained a 1400 bp segment of the 18S rRNA gene and was isolated following [23]. These probes were directly labeled with the Nick-Translation mix kit (Jena Bioscience, Jena, Germany). The 5S rDNA was labeled with ATTO550-dUTP and the 18S rDNA with AF488-dUTP, according to the manufacturer’s manual. The microsatellite sequences (A)_30_, (CA)_15_ and (GA)_15_ were directly labeled with Cy-3 during their synthesis, as described by [24]. These sequences were selected once they are among the most abundant ones in fish genomes [14] and generated well visible hybridization patterns in our experiments. Telomeric (TTAGGG)n sequences were mapped using the DAKO Telomere PNA FISH Kit/FITC (DAKO, Glostrup, Denmark). The FISH experiments followed the methodology described in [25]. Briefly, metaphase chromosome slides were incubated with RNAse (40 μg/mL) for 1.5 h at 37 °C. After the denaturation of the chromosomal DNA in 70% formamide/2× SSC at 70 °C for 3 min, the hybridization mixture (2.5 ng/μL probes, 2 μg/μL C0t-1 DNA, 50% deionized formamide, 10% dextran sulphate) was dropped on the slides, and the hybridization was performed overnight at 37 °C in a moist chamber containing 2× SSC. The first post-hybridization wash was performed with 2× SSC for 5 min at 65 °C, and a final wash was performed at room temperature in 1× SSC for 5 min. Finally, the slides were counterstained with DAPI and mounted in an antifade solution (Vectashield from Vector Laboratories, Burlingame, CA)

### 2.4. Comparative Genomic Hybridization (CGH)

The total genomic DNAs (gDNAs) from male and female specimens of *H. duriventris*, *H. villaboas*, *H. rondoni* and *H. punctata* were extracted from liver tissue by the standard phenol-chloroform-isoamyl alcohol method [26]. It was focused on inter-and intraspecific comparisons, with special emphasis on molecular composition of the putative and multiple sex chromosomes. In the first set of experiments (intraspecific genomic comparisons), the male-derived gDNAs of all species were labelled with Atto550-dUTP and the female gDNAs with Atto488-dUTP, by means of nick translation (Jena Bioscience, Jena, Germany). For blocking repetitive sequences, it was used unlabeled C0t-1 DNA in all experiments (i.e. fraction of genomic DNA enriched with highly and moderately repetitive sequences), prepared according to [27]. The final hybridization mixture for each slide (20 μL) was composed of male- and female-derived gDNAs (500 ng each), plus 25 μg of female-derived C0t-1 DNA from the respective species. The probe was ethanol-precipitated, and the dry pellets were resuspended in hybridization buffer containing 50% formamide, 2× SSC, 10% SDS, 10% dextran sulfate and Denhardt´s buffer, pH 7.0. In the second set of experiments (interspecific genomic comparisons), the gDNA of all male specimens now analyzed, plus the gDNA of *H. punctata* (a species harboring multiple X_1_X_2_Y sex system), were hybridized against metaphase chromosomes of *H. villasboas*. For this purpose, male-derived gDNA of *H. villasboas* was labeled with Atto550-dUTP, while the gDNAs of the other three species were labeled with Atto488-dUTP (*H. duriventris* and *H. rondoni*) or Atto425-dUTP (*H. punctata*) both by means of nick translation (Jena Bioscience, Jena, Germany). In a first slide, the final probe mixture was composed of 500 ng of male-derived gDNA of each *H. villasboas*, *H. duriventris* and *H. punctata* and 10 μg of female-derived C0t-1 DNA of each species. In a second slide, the final probe mixture was composed of 500 ng of male-derived gDNA of *H. villasboas* and *H. rondoni* and 15 μg of female-derived C0t-1 DNA of each species. The chosen ratio of probe vs. C0t-1 DNA amount was based on previous experiments performed in our fish studies [18,28,29]. The CGH experiments followed the methodology described in [30]. 

### 2.5. Microscopic Analysis and Image Processing

At least 30 metaphase spreads per individual were analyzed to confirm the 2n, karyotype structure and CGH results. Images were captured using Olympus BX50 microscope (Olympus Corporation, Ishikawa, Japan), with CoolSNAP and the images were processed using Image Pro Plus 4.1 software (Media Cybernetics, Silver Spring, MD, USA). Chromosomes were classified as metacentric (m); submetacentric (sm); subtelocentric (st) or acrocentric (a) according to [31]. The maps were created using the following software′s: QGis 3.4.3 and Adobe Photoshop CC 2020.

## 3. Results

### 3.1. Karyotypes, C-banding and Sex Chromosomes

**Figure 2:**
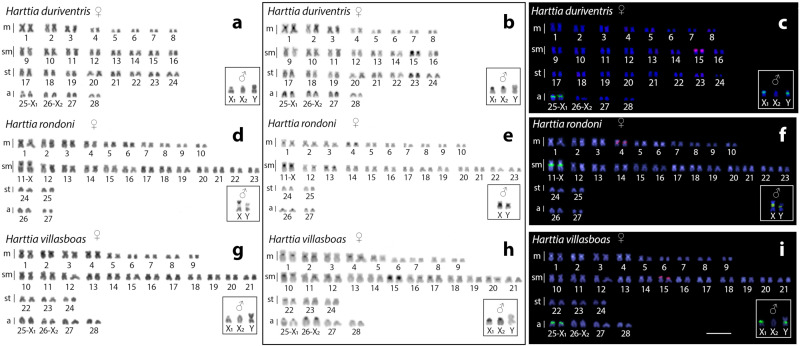
Karyotypes of *H. duriventris* (**a–c**) *H. villasboas* (**d–f**) and *H. rondoni* (**g–i**) arranged by sequentially Giemsa-stained (**a**, **d** and **g**) and C-banded chromosomes (**b**, **e** and **h**) and hybridized with 5S rDNA (red) and 18S rDNA (green) probes after a double-FISH analysis (**c**, **f**, and **i**). Boxes depict the male sex chromosomes. Bar = 5 μm.

*Harttia rondoni* has 2n = 54 chromosomes (20m + 26sm + 4st + 4a) in both sexes. In turn, *H. villasboas* and *H. duriventris* have 2n = 56 chromosomes in the female specimens (18m+24sm+6st+8a in *H. villasboas* and 16m + 16sm + 16st + 8a in *H. duriventris*), but 2n = 55 chromosomes in the male specimens (19m + 24sm + 6st + 6a in *H. villasboas* and 17m + 16sm + 16st + 6a in *H. duriventris*). These specific male karyotypes are due to characteristics X_1_X_1_X_2_X_2_/X_1_X_2_Y multiple sex chromosome systems, where the Y corresponds to a medium-sized m chromosome (Figure 2).

In all species, C-positive heterochromatic bands are found in the pericentromeric region of all chromosomes and in the telomeric region of the acrocentric pairs (Figure 2). In general, C-banding does not identify any chromosome heteromorphism in *H. rondoni* females. However, in males, a slight heteromorphic pattern occurs at the proximal C-positive bands on the long arms of the pair 11 (Figure 2). This points to a likely XX/XY sex chromosome system in this species. On the other hand, no conspicuous heterochromatin accumulation is observed in the Y chromosome of *H. duriventris* and *H. villasboas*.

### 3.2. Chromosomal Distribution of rDNAs, Microsatellite Motifs and Telomeric Repeats

Cytogenetic mapping of the 18S and 5S rDNA sequences showed single sites in corresponding chromosomes of all three analyzed species. Specifically, the 18S rDNA sequences are mapped in the pericentromeric region of the XY chromosomes of *H. rondoni*, where a clear polymorphic state occurs in males (Figure 2). In both *H. duriventris* and *H. villasboas*, these sequences are located in both X_1_ (in females) and in the X_1_ and Y (in males). The statement that these rDNA loci are located on the X_1_ chromosome instead of in the X_2_ is based on available data showing that these sequences are also found in the corresponding X chromosome of the sister species, *H. rondoni*.

**Figure 3:**
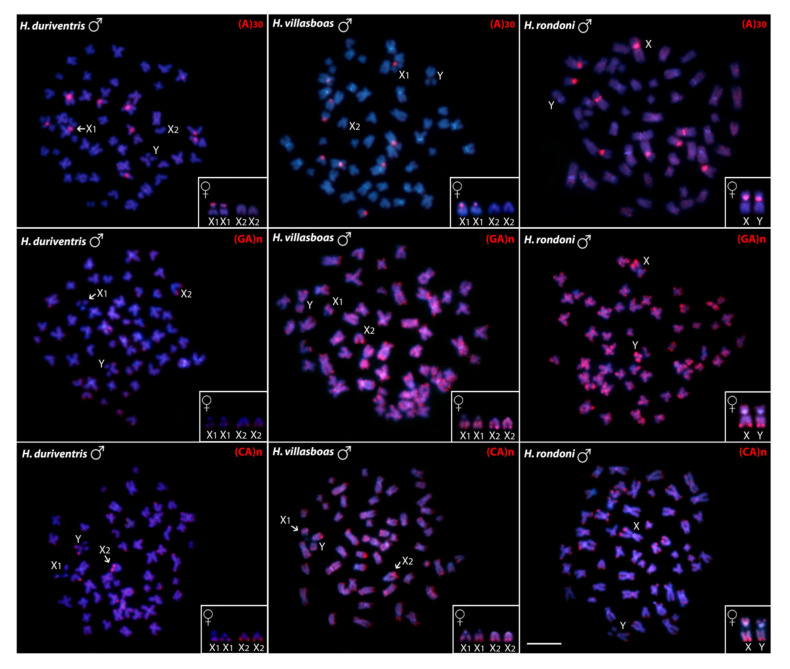
Metaphase chromosomes of males *H. duriventris*, *H. villasboas* and *H. rondoni* hybridized with microsatellite-containing oligonucleotides. Chromosomes were counterstained with DAPI (blue) and microsatellite probes were directly labeled with Cy3 during synthesis (red signals). The female sex chromosomes are shown in boxes. Bar = 5 µm.

The A_(30)_ motif showed hybridization in nine male chromosomes and in ten female chromosomes in all three species, this difference being due to the absence of signals in the Y chromosomes. Besides, *H. rondoni* also presents some small scattered marks in all other chromosomes for this same microsatellite. For the GA_(15)_ probe, small scattered signals occur in the chromosomes of all species, with accumulation on telomeric regions. However, the sex chromosomes also present different accumulation patterns for this microsatellite: on the telomeric region of the X_2_ and the Y chromosomes of *H. duriventris*, on the telomeric regions of the X_1_, X_2_ and Y of *H. villasboas*, and strongly accumulated in the telomeric region of both X and Y chromosomes of *H. rondoni*. Additionally, CA_(15)_ were identified in the telomeric regions of almost all chromosomes, of *H. villasboas*, including the sex ones (Figure 3).

**Figure 4:**
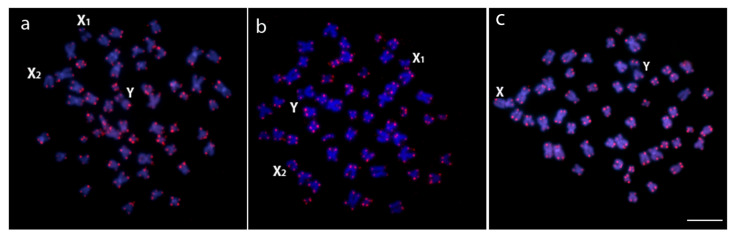
Male metaphase plates of *H. duriventris* (**a**), *H. villasboas* (**b**) and *H. rondoni* (**c**) showing telomeric hybridization signals on both telomeres of all chromosomes. Bar = 5 μm.

FISH with telomeric (TTAGGG)n probe applied to male metaphases of *H. duriventris*, *H. villasboas* and *H. rondoni* revealed hybridization signals on the telomeres of all chromosomes in both species, with no additional interstitial telomeric sites (ITS) (Figure 4).

### 3.3. Comparative Genomic Hybridization (CGH)

#### 3.3.1. Intraspecific Genomic Relationships: Detecting Male-Specific Regions

**Figure 5:**
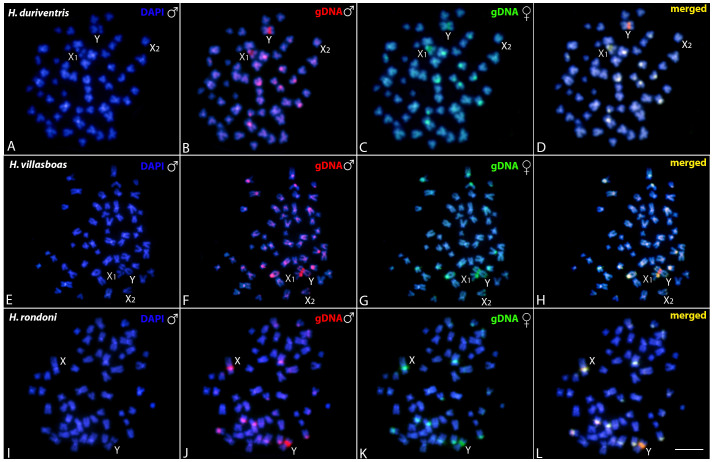
Mitotic chromosome spreads of males *H. duriventris* (**A–D**), *H. villasboas* (**E–H**) and *H. rondoni* (**I–L**) after intraspecific CGH procedures. Male- and female-derived genomic probes were hybridized together for each species. First column (**A**, **E** and **I**): DAPI images (blue); Second column (**B**, **F**, and **J**): hybridization pattern of the male-derived probe (red); Third column (**C**, **G**, and **K**): hybridization pattern of the female-derived probe (green). The fourth column (**D**, **H**, and **L**): merged images of both genomic probes and DAPI staining. The common genomic regions for males and females are depicted in yellow. Sex chromosomes are indicated. Bar = 10 μm.

In all species, CGH procedure failed to detect any conspicuous sex-specific region on male chromosomes (Figure 5). However, a slight binding preference for the male-derived probe to the pericentromeric region of the Y chromosome of *H. villasboas* and *H. duriventris*, and to the Y chromosome of *H. rondoni* was evidenced (Figure 5). Female-derived probe produced only a faint hybridization signal in such regions, while both probes matched equally the large heterochromatic pericentromeric segment in the X_1_ chromosome of *H. villasboas* and *H. duriventris*, and in the X chromosome of *H. rondoni* (Figure 5).

#### 3.3.2. Interspecific Genomic Relationships, Focusing on the Multiple X1X2Y Sex System

**Figure 6:**
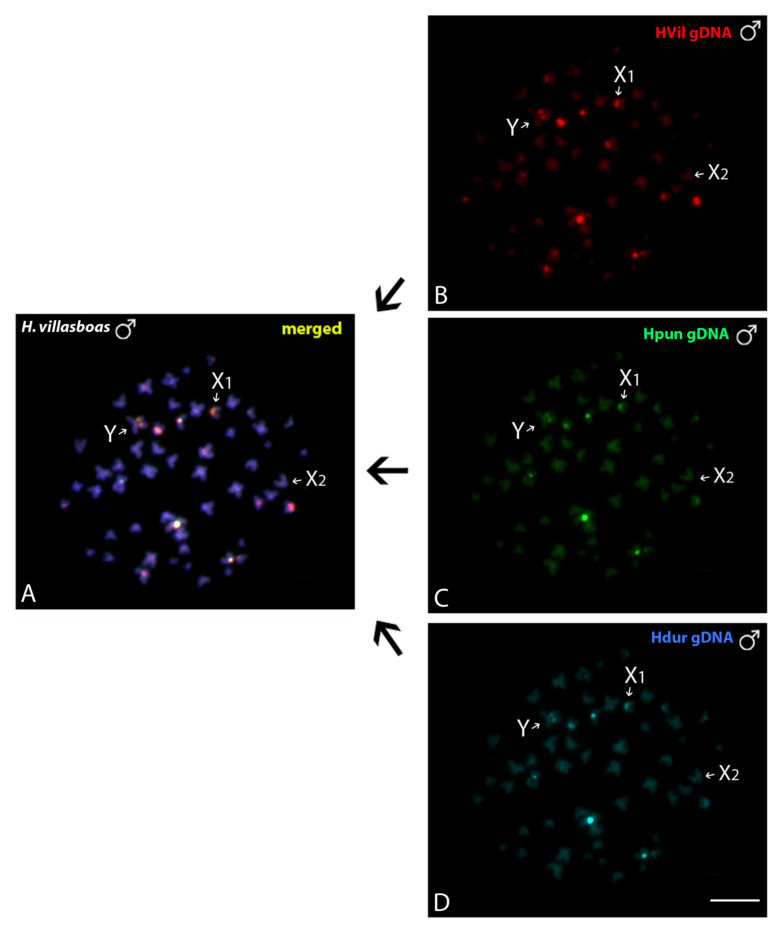
Comparative Genomic Hybridization (CGH) of *Harttia* species bearing X_1_X_2_Y sex chromosome system. Mitotic male chromosome spreads of *H. villasboas* (**A**) hybridized against male-derived genome probes of *H. villasboas* (**B**), *H. punctata* (**C**) and *H. duriventris* (**D**). Sex chromosomes are indicated and the common genomic regions for the three species are depicted in yellow on (**A**). Bar = 10 μm.

When the gDNA of *H. duriventris*, *H. villasboas* and *H. punctata* (all X_1_X_2_Y-species) was compared, no species-specific region in the sex-chromosomes were observed, thus pointing to their common genomic content (Figure 6).

## 4. Discussion

### 4.1. Evolutionary Relationships among Harttia Species

Chromosome data have shown that *Harttia* species, as a whole, presents an extensive variation in the diploid number, karyotype composition, in addition to multiple sex chromosomes in some species [5,6,7,8,10,11,12], the latter corresponding to an uncommon condition among fishes. Here we brought new data for three yet unexplored Amazonian species. If we consider previous available data, it is noticeable that the chromosome numbers of all species now investigated fit into the range of variation previously described for the genus, i.e., 2n = 52♀/53♂ in *H. carvalhoi* [8], and 2n = 62♀♂ in *H. absaberi* [9]. In addition, the general trend of single rDNA locus occurring in rearranged chromosomes is maintained within the genus [13]. However, a striking feature is that they all present new cases of sex chromosomes: an X_1_X_1_X_2_X_2_/X_1_X_2_Y system in *H. duriventris* and *H. villasboas* and a proto or a neo-XY system in *H. rondoni* (discussed below).

The karyotype diversification and morphological patterns are often indicators of the lifestyle of a species [32,33]. Although *Harttia* have a wide geographic distribution in many South American rivers and small streams, their low vagility fosters the fixation of chromosomal rearrangements into small populations, thus promoting chromosomal diversity. Indeed, several other fish species also presenting the same above characteristics, have been evidenced as carriers of a wide variety of karyotypes: *A. fasciatus*, for example, presents karyotypes with 2n = 45, 46, 47, 48 and 50 chromosomes [34,35,36] and *Hoplias malabaricus*, with seven main karyotypes including multiple and simple sex chromosome systems [23,37,38,39]. In Late Cretaceous and Cenozoic, large-scale tectonic events led to changes in river courses and watershed limits, resulting in complex river dynamisms [40], and affecting the distribution of fish populations. For example, the Serra do Cachimbo region (Pará-Brazil), presents highlands that reach 740 m of altitude, divided between Xingu and Tapajós river basins [41]. Located at the northern border of the Brazilian shield, this region has a high number of endemic species such as *Leporinus guttatus* [42], three species of *Lebiasina* genus [43], and *Harttia villasboas* and *H. panara* [41]. This high number of endemic species can be attributed by the high number of headwaters of the Xingu and Tapajós rivers [41]. There, *H. rondoni* and *H. villasboas* have a probable geographic barrier due to a series of waterfalls 40–60 m height, over a 50 m stretch, where *H. rondoni* and *H. villasboas* occur below and above this set of waterfalls, respectively [41,44]. Despite some karyotype similarities that they share, such as the higher number of m and sm chromosomes, a vicariance event may have facilitated the fixation of another series of chromosomal rearrangements in both groups, including the origin of two distinct, but related sex chromosome systems (a proto or- neo-XY system in *H. rondoni* and a multiple X_1_X_1_X_2_X_2_/X_1_X_2_Y system in *H. villasboas*). The generation of multiple sex chromosome systems usually involves centric fusions or fissions events and may retain vestiges of interstitial telomeric sequences (ITS) [45]. Our chromosomal mapping of telomeric sequences only reveal the expected terminal signals, without any ITS in the three species. However, it is also known that Robertsonian rearrangements can also lead to the loss or reduction of sequences close to the chromosomal breakpoints [45], and it is likely that this particular condition is responsible for the absence of ITS, as well as of microsatellite sequences and C-positive heterochromatin that, although identified on the X_1_ and X_2_ chromosomes of *H. duriventris* and *H. villasboas*, they are missing in the Y chromosome. 

**Figure 7:**
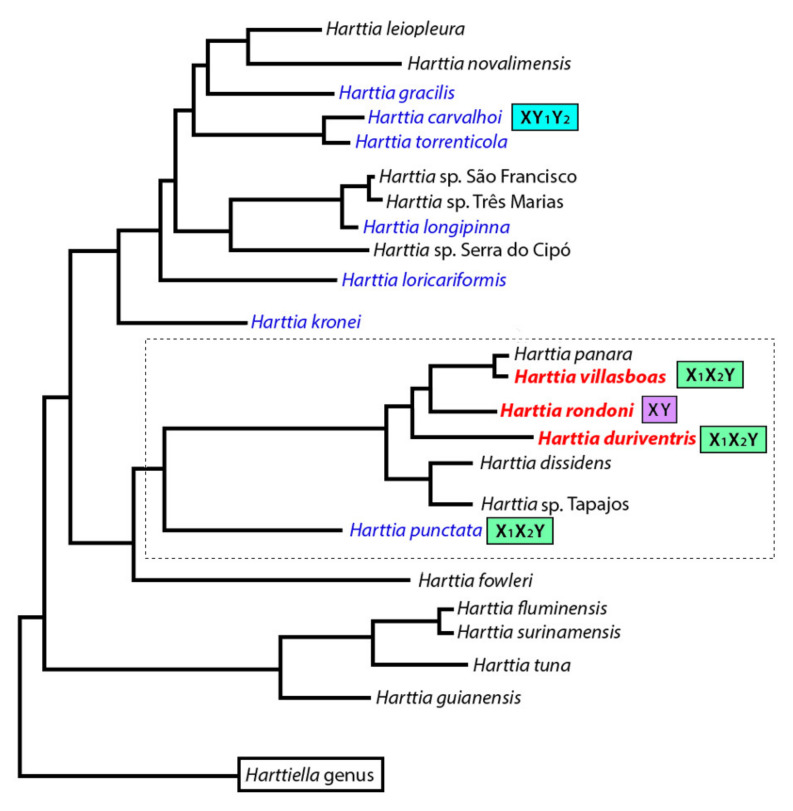
Adapted phylogenetic tree for the genus *Harttia*, based on the molecular-phylogenetic data generated by Covain [19]. Previous and now cytogenetic analyzed species are indicated in blue and red, respectively. The multiple X_1_X_2_Y (green boxes) and XY_1_Y_2_ (blue box) sex chromosome found in *Harttia* is also indicated, together with the putative proto or neo XY system (purple box). Species from northern Brazilian region are highlighted with the dotted line.

Blanco [13] discussed the split of *H. punctata* from a southeast clade with the origin of its multiple sex chromosome system being a characteristic determinant, probably originated from an ancestor with 2n = 58 chromosomes without a differentiated sex chromosome system. Now, one specific chromosomal pair present in *H. rondoni* shares similar patterns to the X_1_X_2_Y sex chromosomes present in the other closely related species, opening two main scenarios: (i) a proto-XY hypothesis: The X and Y chromosomes differ only slightly due to the amplification of repetitive sequences (major rDNAs) on the X chromosome, thus representing an early stage of differentiation. As all its closely related species until now studied have sex chromosomes (Figure 7, dotted line), we could hypothesize that this 2n = 58 ancestor have also carried a proto-XY sex system, as is the case of *H. rondoni*. Afterwards, chromosomal fissions on the X chromosomes may have created the X_1_ and X_2_ chromosomes present in *H. punctata*, *H. duriventris* and *H. villasboas*. (ii) A Neo-XY hypothesis: chromosomal fusions involving the ancestral X and Y chromosomes and a pair of autosomes could create such a neo-XY sex system, leading to the reduction of the 2n from 56 (present in the sister species) to 54 in *H. rondoni*. Besides, the bigger size of the neo-X chromosome in comparison with the Y and the loss of a A(n) rich region closer to the ribosomal loci in the later support this hypothesis. Among fishes, some cases of neo-XY sex chromosomes emerging from X and/or Y-autosomal translocations have been also observed [46,47,48]. Neo-sex chromosomes may not necessarily lead to the emergence of multiple sex chromosomes, as equal addition of autosomal segments to both sex chromosomes generates neo-XY or neo-ZW systems. Complementary studies, focusing on whole chromosome painting and in the genomic organization of these sex chromosomes, are necessary to clarify these two abovementioned hypotheses.

### 4.2. The Genus Harttia as A Repository of Multiple Sex Chromosome Systems

Teleost fishes represent one of the most diverse groups in terms of sex determination and differentiation [49,50]. Heteromorphic sex chromosomes are identified in about 5% of so far analyzed species [51], mostly corresponding to simple systems, the XX/XY being the most frequent one [52]. Multiple sex chromosomes occurs in a much lower number and, according to Pennel [53], 47 of such occurrences had been so far recorded, encompassing five different kinds of systems, named ♀X_1_X_1_X_2_X_2_/♂X_1_X_2_Y, ♀XX/♂XY_1_Y_2_, ♀X_1_X_1_X_2_X_2_/♂X_1_Y_1_X_2_Y_2_, ♂ZZ/♀ZW_1_W_2_ and ♂Z_1_Z_1_Z_2_Z_2_/♀Z_1_W_1_Z_2_W_2_, the first of them being the most prevalent one [54]. Here, a marked feature of both *H. villasboas* and *H. duriventris* karyotypes is an X1X2Y sex chromosome system. Such occurrences, together with the two previous multiple systems described for *H. punctata* (X_1_X_2_Y) and *H. carvalhoi* (XY_1_Y_2_), makes *Harttia* the genus with the most abundant number of multiple sex chromosome systems up to now identified among fishes [53].

It is known that Y-A fusions are the most common rearrangements related to the origin of X_1_X_2_Y systems [53] and it is likely that this is also true for both *H. duriventris* and *H. villasboas* cases. But the find of the concomitant proto/neo XY-system in the sister species, *H. rondoni*, adds a relevant question in this evolutionary puzzle. In such system the sex pair differ only slightly by the accumulation of repetitive sequences in only one of the chromosomes, i.e., the Y one. This heteromorphism was not always clearly detected on the pattern of the C-positive heterochromatin, but it was evident after the 18S rDNA mapping and the intraspecific CGH experiments. This scenario alone could raise doubts about the existence of such a proto-XY system, reinforcing the probable neo-origin to this chromosomal sex system. However, when the chromosomal pattern of *H. rondoni* is compared with those of *H. villasboas* and *H. duriventris*, it is possible to establish the karyological relationships among these species supporting the real occurrence of the putative sex chromosomes in *H. rondoni*, and the origin of the multiple X_1_X_2_Y sex chromosome systems in the two latter species. In fact, comparative genomic hybridization (CGH), plus chromosomal mapping of microsatellites and rDNAs repeats highlight that these three *Harttia* species share the same distribution pattern in the sex chromosomes. Although *H. punctata* also presents an X_1_X_2_Y system, its rDNA distribution includes both 5S and 18S sequences on the sex chromosomes [11,13], which is different from *H. duriventris* and *H. villasboas* where only 18S motifs occur on them, probably by a translocation of the 5S rDNA motif, present in the X_1_ of *H. punctata*, to autosomal chromosomes during lineage diversification in *H. duriventris*, *H. villasboas* and *H. rondoni*.

Meaningly, a close association between microsatellites, rDNAs and multiple sex chromosomes has been reported for many fish taxa [55,56,57,58,59], which highlights the probable role of these sequences in the genesis of such systems. Despite our CGH data do not reveal any conspicuous Y-specific region, neither in both Y chromosomes of *H. duriventris* and *H. villasboas*, nor in the proto/neo-Y of *H. rondoni*, a slight binding preference for the male-derived probe occur at the pericentromeric region of all these chromosomes (Figure 5). Potential effects of repetitive DNA accumulation on recombination rate have been considered [60], what could explain their initial accumulation on the sex chromosomes. When sex chromosomes stop recombination, repetitive sequences are predicted to have a rapid accumulation on them [61], and microsatellite repeats seem to play a key role as “early colonizers” in their differentiation [24,62]. Numerous examples in animals and plants document a massive and differential accumulation of such small motifs in sex-specific chromosomes, particularly in simple (XY or ZW) systems [24,56,63,64,65,66,67]. However, most X_1_X_2_Y systems lack substantial differentiation in the neo-Y, since the accumulation of repetitive DNAs (= large blocks of heterochromatin) would impair the proper pairing of the neo-sex chromosomes into a stable trivalent form, thus disturbing the meiotic process [68]. Here, no significant differences in microsatellites distribution could be identified concerning autosomes and sex chromosomes of the *Harttia* species. In general, while (A)_30_ sequences presented a strong accumulation pattern in the pericentromeric regions of some chromosomal pairs, both (CA)_15_ and (GA)_15_ displayed a widespread distribution pattern, with preferential accumulation in some telomeric regions (Figure 3). Besides, as evidenced by C-banding and CGH experiments, all the X_1_, X_2_ and Y chromosomes do not accumulate repetitive sequences, and the Y chromosome of *H. rondoni* is even missing (A)_30_ sequences probably lost after the chromosomal rearrangements related to its genesis. Thus, the recombination suppression in multiple sex chromosome system is a question that deserves to be better clarified.

According to the most updated phylogeny proposed for the genus *Harttia* [19]. *H. duriventris*, *H. villasboas*, and *H. punctata*, all harboring multiple X_1_X_2_Y sex system, are evolutionary related and belong to the same major clade. In accordance with, our CGH experiments showed that they sex chromosomes share genomic content, thus pointing to their relatedness and to a probable common origin. This is a characteristic that, in general, is not found among teleosts species. Although some *Megaleporinus* [69], *Parodon* [70] and *Characidium* species [71], as well as the whole genus *Triportheus* [66,72], have their sex chromosomes with a common origin and differentiation, this is an exceptional scenario [73]. In fact, an independent origin is more commonly found, even among congeneric species [49,52,74,75,76,77,78]. According to Schartl [79], such a situation could be explained by the emergence of sex-determining genes on different chromosomes which, by mutation, would promote male or female development and giving rise to new sex chromosomes. Next steps, implying finer-scale approaches such as Zoo-FISH experiments with whole chromosome painting (WCP), coupled with recent genome sequencing procedures, will shed more light on this issue, especially in the recent or ancient origin of the XY system of *H. rondoni* and its significance to the group.

## 5. Conclusions

Chromosomal data of the Brazilian northern *Harttia* species (*H. duriventris*, *H. villaboas*, *H. rondoni* and *H. punctata*) support the common origin for their sex chromosome systems. Additionally, the results also allowed us: (i) to track their evolutionary relationships with other *Harttia* species, adding new light on their relatedness inside the genus; (ii) to describe two new multiple sex chromosomes systems of the X_1_X_2_Y type; and (iii) to highlight a proto or neo-XY system in *H. rondoni* and its close association with the aforementioned multiple sex systems. The remarkable variation on karyotypic organization, in addition to the frequency and different types of sex chromosomes systems inside the genus, makes *Harttia* an useful model for evolutionary studies among fish focusing on karyotype differentiation and sex chromosomes evolution.

## Figures and Tables

**Table 1:** Collection sites and samples size (N) of the species analyzed.

Species	Locality	N
1. *H. duriventris*	Parauapebas River, Canaã dos Carajás-PA (Brazil) (6°30’06.5’’ S 50°02’35.3’’ W)	08♀, 07♂
2. *H. villasboas*	Curuá River, Cachoeira da Serra-PA (Brazil) (8°44’09.0’’ S 54°57’46.0’’ W)	34♀, 38♂
3. *H. rondoni*	13 de Maio River, Cachoeira da Serra-PA (Brazil) (8°38’53.0’’ S 55°01’41.0’’ W)	15♀, 14♂
4. *H. punctata*	Itiquira river, Formosa—GO (Brazil) (15°19’25’’ S 47°25’26’’ W)	10♀, 12♂
